# Correction: Bastyte et al. The Association of Vitamin D Receptor Gene Polymorphisms with Vitamin D, Total IgE, and Blood Eosinophils in Patients with Atopy. *Biomolecules* 2024, *14*, 212

**DOI:** 10.3390/biom16060775

**Published:** 2026-05-26

**Authors:** Daina Bastyte, Laura Tamasauskiene, Ieva Stakaitiene, Rasa Ugenskiene, Brigita Gradauskiene (Sitkauskiene)

**Affiliations:** 1 Department of Immunology and Allergology, Lithuanian University of Health Sciences, LT-50161 Kaunas, Lithuaniabrigita.gradauskiene@lsmu.lt (B.G.); 2 Laboratory of Immunology, Department of Immunology and Allergology, Lithuanian University of Health Sciences, LT-50161 Kaunas, Lithuania; 3 Department of Genetics and Molecular Medicine, Lithuanian University of Health Sciences, LT-50161 Kaunas, Lithuania

There was an error in the original publication [1]. This error was due to an unintentional typing mistake in the calculation during data entry. After identifying the issue, all values were carefully recalculated and corrected accordingly. Corrections have been made to the following: Abstract; Section 3.3, paragraph; Table 3; Discussion Section. 

The corrected content is availalble below:

**Abstract:** In addition, a decreased amount of vitamin D was associated with atopic diseases such as allergic asthma (OR 0.58; 95% CI: 0.372–0.908, respectively, *p* < 0.05).

*3.3. Relationship between VDR Polymorphisms and Vitamin D Level*

The comparison of vitamin D blood levels in the studied groups is demonstrated in Table 3. Binary logistic regression analysis showed that decreased vitamin D levels were associated with allergic asthma (Table 3). 

**Table 3:** Atopic diseases and vitamin D level.

Vitamin D Level (ng/mL)	Groups *n* (%)		OR	95% CI	*p*-Value
<20 20–30 30–50	Atopic dermatitis (*n* = 45)	Control (*n* = 81)			
	20 (44.4%)	31 (38.3%) 32 (39.5%) 18 (22.2%)	1.076	0.481–2.410	0.858
	18 (40.0%)				
	7 (15.6%)		0.758	0.439–1.308	0.319
	Allergic asthma (*n* = 77)				
	51 (66.2%)		0.304	0.144–0.642	0.002 *
	16 (20.8%)				
	10 (13.0%)		0.581	0.372–0.908	0.017 *

* Significant difference. OR: odds ratio. CI: confidence interval.

**4. Discussion**

The results of the present study indicated a significant association between decreased vitamin D levels and allergic asthma. 

The authors state that the scientific conclusions are unaffected. This correction was approved by the Academic Editor. The original publication has also been updated.
